# Creating a Tobacco Cessation Program for People with Disabilities: A Community Based Participatory Research Approach

**DOI:** 10.4172/2155-6105.1000204

**Published:** 2014-12-21

**Authors:** Jamie L Pomeranz, Michael D Moorhouse, Jessica King, Tracey E Barnett, Mary Ellen Young, Vani Simmons, Thomas Brandon, Nichole Stetten

**Affiliations:** 1 University of Florida, Newberry, United States; 2 University of South Florida, United States

## Abstract

**Introduction:**

Smoking is the single most preventable cause of morbidity and mortality, accountable for one out of every five fatalities in the United States annually. Fifty million Americans (22%) suffer from some form of disability, with evidence suggesting that smoking rates within the disabled community are double that of the general population.

**Methods:**

The purpose of this study was to develop a tobacco cessation program designed by and for people with disabilities (PWD). Limited research data regarding tobacco interventions suggest that both adapting treatment methods and developing novel approaches may be effective in establishing cessation programs for low-income populations. Community-Based Participatory Research (CBPR) was conducted to develop a tobacco cessation group treatment program for PWD. Consumers with disabilities who use tobacco were recruited from a large population of PWD utilizing services at multiple centers for independent living (CIL) within North Central Florida.

**Results:**

Following qualitative interviews, multiple Community Advisory Board (CAB) meetings, and expert panel review, the tobacco cessation program was modified across several areas including: updating epidemiological data, decreasing text density, adding personal vignettes from PWD, adjusting for person-first language, adding disability-specific issues, and incorporating appropriate counseling strategies.

**Conclusions:**

Study findings suggest that CBPR-based methods are useful when developing tobacco cessation programs for persons with disability. Forty-two changes were recommended for the resulting LIFT Curriculum. Next steps include pilot testing the curriculum among individuals with disability and comparing results to a standard tobacco cessation curriculum.

## Introduction

Tobacco use is the leading cause of preventable death in the United States (USA), accounting for approximately 480,000 deaths domestically [1] and more than 5 million deaths worldwide each year [2]. While the health risks associated with tobacco use have been well documented, and rates have steadily declined since the mid-1960s, nearly one in five adults in the USA continue to use tobacco [1]. Perhaps more concerning, tobacco use among certain populations remains disproportionately high, and little, if any, efforts have been initiated to address this discrepancy.

The Centers for Disease Control and Prevention (CDC) estimates that approximately 24.2% Americans experience some form of disability [3], and evidence suggests that people with disabilities (PWD) are 50% more likely to smoke than their non-disabled counterparts [3,4]. It is important to note that disability definitions are often dependent on the organization providing services and support to this population. For example the Social Security Administration (SSA) defines disability as the inability to work due to a medical condition that lasts at least one year or results in death [5]. In addition to SSA, the Americans with Disabilities Act (ADA), which prohibits discrimination while promoting equal opportunity for persons with disabilities, provides a definition that includes substantial limitations in one or more major life activities. These activities relate to self-care, performing manual tasks, walking, physical functioning, and executive functioning [6]. A more recently developed definition of disability has been issued and published by the World Health Organization (WHO) and is based on a classification using a Biopsychosocial model known as the International Classification of Functioning, Disability, and Health. According to WHO, disability is a complex phenomenon that incorporates both the medical and social models of disability [7].

When compared to people without disabilities, people with disabilities (PWD) are more likely to have ever smoked, be current smokers, have fewer quit attempts, and smoke more cigarettes per day [3]. Additionally, while PWD encounter the same health conditions as the general population, they may acquire these conditions at an earlier age and have additional health consequences [8]. Consequently, tobacco cessation for PWD is particularly important given the negative impact of tobacco on their medical conditions.

Schroeder and Morris found that the increased rate of smoking among individuals with disability is due to biological, psychosocial, cultural, and tobacco industry related factors, as well as lack of appropriate cessation interventions [9]. Given that PWD generally have lower education and socioeconomic levels [3], it is safe to assume that a lack of financial resources, health insurance, or health literacy may prevent them from obtaining the same cessation support as their non-disabled counterparts. Likewise, individuals with cognitive, affective, or sensory impairments may have difficulty obtaining, understanding or remembering cessation materials [8,10-12].

Recent research has also shown that national organizations that support PWD may be ill-equipped to address tobacco cessation. Moorhouse et al. [13] surveyed 431 Center for Independent Living (CIL) directors to assess the priority of tobacco cessation and resources available for tobacco cessation at their centers. Although tobacco cessation was identified as the second highest health priority by Directors, respondents reported that tobacco cessation was among the most inadequately delivered services at CILs. Moreover, less than 5% of Directors reported having a way to identify CIL clients who use tobacco, less than a quarter (23.4%) reported having self-help cessation materials available on site, few (1.6%) reported having an individual cessation program, and none reported offering tobacco cessation groups [13].

Finally, although PWD face a series of unique challenges relative to tobacco cessation (e.g., unable to sit or participate for a designated amount of time, unreliable transportation, personal care attendant dynamics, and inability or limited capacity to participate in physical activity), intervention efforts are typically developed for the general population and fail to address or consider these challenges. PWD represent a large proportion of the USA population and yet have been relatively understudied in terms of health promotion and disease prevention activities [14,15]. To date, there has been no known tobacco cessation intervention tailored to PWD. Therefore, the purpose of this article is to describe the multiple steps involved in the development of the Living Independently from Tobacco (LIFT) curriculum—a tobacco cessation program designed specifically for PWD. Findings are presented within a community based participatory research framework.

## Methods

**Figure 1:**
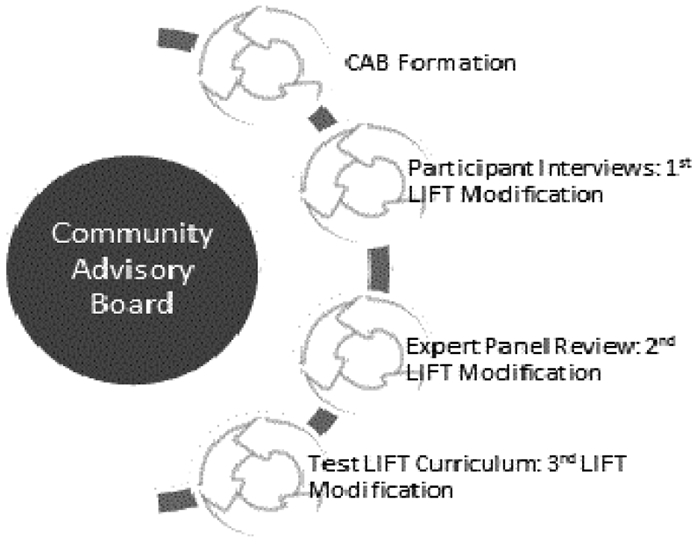
CBPR Process.

The LIFT curriculum was developed through an iterative process consistent with the tenets and practices of community based participatory research (CBPR) [16-18]. The process of developing the curriculum is depicted in Figure 1 and included the following steps: Step 1: development of the community advisory board (CAB) and procedures for community involvement; Step 2: qualitative interviews with PWD regarding acceptability of cessation strategies; Step 3: CAB review of initial draft of curriculum; and Step 4: expert panel review of revised version of curriculum. Participants at each step of the process completed informed consent. This study was approved by University of Florida IRB01.

For the purposes of this study, we used the definition of disability used by the CILs. Throughout the study, as detailed below, we partnered with the North Central Florida CIL (CILNCF). The CILNCF serves over 2,500 PWD from 16 counties in the North Central Florida region. The CILNCF is part of a national organization of Centers for Independent Living (CIL) that support PWD in overcoming barriers such as low income and limited access to rehabilitation services. The CIL, which was founded by PWD who understand the challenges faced by their consumers, is a national leader in supporting PWD in their efforts to lead independent lives, with over 500 locations throughout the USA [19]. All CILs require that 51% of staff and 51% of Board of Director members have a disability. The overall goal of CILs is to reduce environmental barriers and empower individuals with disabilities to overcome such barriers [20].

### Step 1 Procedures:Development of Community Advisory Board (CAB)

The development of the LIFT curriculum followed a CBPR approach. Cargo and Mercer [16] define ‘participatory research’ as “an umbrella term for a school of approaches that share a common philosophy of inclusivity and of recognizing the value of engaging in the research process those who are intended to be the beneficiaries, users, and stakeholders of the research…”. The CAB consisted of university staff and community members with disabilities (Table 1). All participants with disabilities were recruited from the CILNCF. The primary sources of recruitment were word of mouth and flyers. The researchers actively sought to recruit a diverse sample based on disability. Most fundamental to a CBPR approach is to establish a genuine collaborative partnership that includes the skills and resources of all partners in each step of the research process. Consistent with CBPR approaches, PWD were not only study participants, but they served as active research consultants who provided direction for the project. Because the goal of this study was to develop a sustainable tobacco cessation program for and facilitated by PWD, the researchers considered the disability perspective throughout the research process. Thus, PWD were instrumental in the continuous modification and adaptation of the research method, as well as the final curriculum and current manuscript.

### Step 2 Procedures:Qualitative Interviews

Semi-structured qualitative interviews were conducted with a convenience sample (n=10) of PWD from the CILNCF. Recruitment continued until a variety of disabilities was represented and saturation was reached. The final sample consisted of four women and six men with varying disabilities (Table 1) who completed an interview that ranged from 20 to 50 minutes, depending on participant’s responses. The topics covered in the interview included the participant’s tobacco history, likes and dislikes about using tobacco, number of and experience with previous quit attempts, importance of quitting smoking, thoughts about people with disabilities using tobacco, what should be included in a cessation program for individuals with a disability, barriers to quitting smoking for people with disabilities, and ideal location to participate in a cessation program. The interviews were conducted by the research team at the CILNCF.

All interviews were audio recorded. Each audio file was transcribed to generate a typed verbatim transcript of each interview. Data analysis was conducted using NVivo^®^ Qualitative Software. NVivo^®^ provides the researcher with a means for handling extensive narrative data, such as interview transcripts, and for browsing text, coding data into categories, establishing the definitions and properties of the categories, and modeling the relationships among categories [21]. Three members of the research team independently coded the transcribed interviews. Any discrepancies were discussed until consensus was reached.

### Step 3 Procedures: CAB Review of the Curriculum

To engage PWD in the development process, a Community Advisory Board (CAB) was formed. The CAB was comprised of males and females with disabilities who could provide insight into the barriers associated with tobacco treatment (Table 1). We specifically sought to recruit members representing multiple disabilities, including both physical and mental disabilities. CAB members were asked to review an existing tobacco cessation group program manual, Quit Smoking Now (QSN), developed by the Florida Area Health Education Center and ex-smokers. QSN is a program designed by former smokers to help people living in Florida quit using any tobacco product. The curriculum incorporates the philosophy of both the CDC’s Best Practices for Comprehensive Tobacco Control [22] and the Clinical Practice Guidelines for Treatment of Tobacco Use and Dependence [23]. Specifically, the program includes six sessions designed to help tobacco users identify goals, manage their addiction, set a quit date, prevent relapse, develop a plan after treatment, and learn effective ways to continue with the recovery process. CAB members were asked if the program: 1) appeared to be applicable to individuals with disabilities, 2) could be used for individuals across all disability groups, 3) needed modifications to become more applicable for PWD, and 4) could lead to continued tobacco cessation for this unique population.

### Step 4 Procedures: Expert Panel Review

Data identified from the CAB and qualitative interviews were presented to a tobacco expert panel for feedback regarding development, modification and verification. Seven expert panel members were identified based on extensive work and knowledge of tobacco cessation, and invited to review the curriculum. The panel included three psychologists, one general physician, one psychiatrist, and the leader of the CAB to maintain continuity (Table 1). Experts were asked to comment on the applicability of the feedback provided by the CAB towards creating a tobacco cessation group program for PWD.

Hard copies of the manual were shipped to each expert for review. The experts first went through the manual and edited, noted questions, and made suggestions throughout the book. Two weeks after receiving the manual the panel met by conference call and discussed each of the suggestions in detail to achieve consensus among the experts regarding which changes were imperative. At the conclusion of the virtual meeting, the expert panel members returned their manuals to note all detailed suggestions throughout each manual.

## Results

Following qualitative interviews, multiple CAB meetings, and expert panel review, the tobacco cessation program was modified across several areas including: updating epidemiological data, decreasing text density, adding personal vignettes from PWD, adjusting for person-first language, adding disability-specific examples, and incorporating appropriate counseling strategies and other pertinent information within a facilitator guide. Table 2 highlights each of the proposed changes, including whether the change was suggested during the qualitative interview stage, CAB meetings or expert review.

### Qualitative Interview Results

The 10 qualitative interviews helped provide the context for understanding tobacco usage among persons with disabilities. Furthermore, the participants provided recommendations for making a cessation program more applicable for people with disabilities through highlighting appealing components of a quit program and barriers to quitting specific to PWD.

#### Logistics:

Participants were supportive of a tobacco cessation program at the CIL for a number of reasons. Some participants listed a location on the bus route as a priority. Other participants requested a location that people with disabilities frequent because “it is part of their routine already” (Participant 2). A reoccurring theme was that of a familiar location, which for many people with disabilities, is the CIL. Participant 5: “Here is more comfortable. I know where it is. I know how to find it.” Participant 9 went on further to say “when I feel down and very unsatisfied, I come by here, sit down … this place helps a lot. This place here is my second home.”

Another change suggested by participants was to increase the total number of sessions, the frequency of sessions per week and duration of sessions. Several individuals remarked on the importance of keeping busy, particularly for people with disabilities. As participant 7 mentioned, a major barrier in successful cessation is having a “disability that prevents from working or being active”.

#### Manual

Through the qualitative interviews, participants provided information regarding how to adapt the curriculum to be more relevant to people with disabilities. Participant 8 introduced the idea of focusing less on cigarettes and more on stressors and stress management: “the thing I noticed from being able bodied to disabled is I was a very independent person. I can’t be that now. Sometimes that frustrates the heck out of me.” Not only addressing managing daily life stressors, but, as Participant 4 said “deal with the stress of disability”.

Additional considerations include addressing medication interactions with nicotine replacement therapy: “I find that I could not use the patch, relating to the medication I took” (Participant 3) as well as providing specific information regarding smoking’s effect on the body, focusing on interaction between smoking and disability.

#### Facilitator Guide

Many of the reasons for smoking and barriers to quitting were similar to those among people without disabilities. These factors will also be considered within the facilitator guide.

Several participants described peer pressure to use tobacco at first and continuing to use tobacco to ‘fit in’: “I wanted to be like the rest of them. I wanted to hang out in the crowd. Everybody was puffing on a cigarette… a lot of people that are disabled want to blend in with the crowd” (Participant 1).

Five participants described tobacco use to relieve stress; others indicated cravings, pleasure, boredom, or social situations as reasons for usage: “It just eases away some of the pressure that is on me” (Participant 1). Seven out of the 10 persons with disabilities interviewed indicated low self-efficacy for quitting in response to the question “How confident are you that you can quit?” Another seven listed health reasons as one of the reasons to quit: “I have severe COPD. I have a spot on my lung. I have lung cancer” (Participant 8). However, those health reasons also were connected to participants’ continued tobacco use: “A lot of time when I think about my illness I smoke even heavier” (Participant 5).

The qualitative data supported a foundational premise for the study: people with disabilities, even when faced with chronic conditions that lead to significant disability, continue to use tobacco and have little confidence in their ability to quit.

### CAB Review

Throughout each review process, the CAB members provided a number of specific modifications to the curriculum and updates to the facilitator guide.

#### Manual

A common theme was to address characteristics relevant to PWD: “some examples will need to be re-written to be more inclusive” (CAB 3), such as recommending participants walk for stress management. Another suggestion was “having tapes for those with visual difficulties and interpreters for deaf/hard of hearing participants” (CAB 2). Manual format also received considerations. Several members suggested increasing the font sizes to accommodate individuals with visual or functional limitations and to improve readability for those with cognitive limitations.

#### Facilitator Guide

Additionally the CAB had suggestions for facilitators. One CAB member addressed the common need to hold onto the habit among smokers with disability: “frequently I have heard people with disability regard their smoking habit as ‘all I have left’ or ‘the only thing left that I like to do and can do’, maybe find a way to confront that and dispel” (CAB 6). Another suggestion was to be cognizant of the possibility that “tobacco usage was a coping mechanism, during particularly stressful times. Such as, a person with MS (Multiple Sclerosis) experiencing a relapse of symptoms, which can be sudden and very debilitating, this may cause a fallback on tobacco usage” (CAB 4).

### Expert Panel Results

The expert panel members provided a detailed scientific review and suggestions throughout the manual. Their feedback brought the manual up-to-date in all areas of best practices regarding tobacco cessation. They updated side effects of certain medications that were missing (e.g., for Varenicline (Chantix) “the most common side effects are nausea (30%) and abnormal dreams (20%))” while also making suggestions for reducing the reading level (e.g., substituting scientific language with text more appropriate for a lay audience). Experts also made several suggestions regarding the visual appeal of the curriculum such as adding more graphs and visuals and reducing the potentially burdensome heavy text.

One particular area that received ample expert advice was the way the quit plan was presented. The experts felt the existing quit plan was too “prescriptive” and that the participants should participate more in making the quit plan unique to work best for them. They noted barriers in the prescribed quit plan, such as implying that a physician appointment is needed to get started. This language was softened and revised to be a suggestion that participants make an appointment with their physician to discuss quitting smoking, and any potential effects for medications they might experience. Some of the suggestions were relevant to the accompanying facilitators guide. For instance, the experts warned that participants might ask about alternative treatments, such as acupuncture, hypnosis, or e-cigarettes and thus this information was included in the facilitators guide along with tools to guide the participant back to the proven and recommended cessation methods. The experts also noted that participants might ask about combination medication therapies. To incorporate this, the part of the quit plan regarding medications was modified to “select all” medications that a participant might be interested in trying. A final suggestion for medications was that facilitators have NRT samples ready and on-hand at group sessions so participants can see them and view demonstrations regarding their use. Specific recommendations were incorporated into the manual that should also be discussed, such as “rotate the patch” as this reduces skin irritation.

While the experts provided detailed feedback for the manual, they also made notes regarding the overall program. For example, they suggested that during orientation the group be solicited for rules regarding how to best run a group that is beneficial to all members. A new suggested addition was the rule “only one person speaks at a time” be added to the manual and also discussed during the orientation. This will also be expanded in the facilitators guide for tricks to manage problems with multiple persons talking or a dominant person in a group. Finally, the original manual included quit line resource information up front, in the quit plan section. An expert suggested this might be confusing given they are coming to a cessation group. The quit line information was thus moved to the back of the new manual, in a section for suggested places to continue getting support. This should now be seen as a place to continue obtaining support after the program has concluded.

## Discussion

According Kerr et al. [24], the body of evidence related to tobacco cessation for PWD is limited. In fact, states with high prevalence of tobacco use such as Florida do not even specifically target individuals with disability who use tobacco [25]. As pointed out by Borelli and colleagues [26], tailored programs that target sub-populations may be more effective for special populations than tobacco treatment programs intended for the general population. Surprisingly, however, an evidence-based disability-tailored tobacco cessation program does not exist.

Past CBPR prevention and intervention research tailored to PWD have been more effective in health behavior change compared to tradition health promotion programs, further validating the need to create a tobacco cessation program for PWD [27-30]. To our knowledge, this study represents the first CBPR study aimed at targeting a tobacco cessation program to PWD. The present study resulted in comprehensive tobacco cessation group program curriculum for PWD. Consistent with CBPR methodology, the LIFT curriculum was designed in collaboration between the research team and the community of individuals with disabilities who were either current or former tobacco users. The resulting curriculum incorporated 42 adaptations, making the LIFT more accommodating for PWD. These changes included logistics such as program location, tools used by participant to facilitate success, and recommendations for a facilitator of a tobacco cessation program for PWD.

In order to make the curriculum information more relatable, case scenarios were developed that address tobacco use among several different types of disabilities. Individuals with different types of disabilities present both physical and cognitive limitations. The resulting curriculum is thus targeted towards PWD, with additional guidelines for facilitators on how to further tailor the program.

This study is not without limitations. Specifically, data were collected from a relatively small sample and might not be representative of all individuals with disability. However, we do feel saturation was reached based on feedback from the CAB members, expert panel, and the analysis of the qualitative interviews. Additionally, while our sample of individuals with disability consisted of individuals with cognitive and physical impairments, each of our participants were capable of completing paperwork and participating in the interview or review process. Therefore, while efforts were made to make the manual as accessible as possible, the resulting curriculum might not be adequate for individuals with severe cognitive disability.

We utilized a CBPR approach to take a mainstream tobacco cessation curriculum and modify it for people with disabilities. Through creation of a community advisory board to assist throughout the process, qualitative interviews with individuals with varying disability, CAB review meetings, and expert panel review, we identified 42 areas for adaptation. These changes in logistics, the manual, and the facilitator guide resulted in a program that is more applicable for people with disabilities. Future research includes testing the LIFT curriculum and comparing results to the standard Quit Smoking Now program.

## Figures and Tables

**Table 1:** Composition of the Participants (CAB, Interview of PWD, and the Expert Panel).

Stakeholders	Age	Sex	Race	Expertise	Disability
CIL-Affiliated CAB Members	48	M	W	*Director of Independent Living Center for People with Disabilities	Spinal Cord Injury
	50	F	W	Research Scientist, Qualitative Research, Disability Research	Muscular Dystrophy
	53	F	B	Administrator for Independent Living Services People with Disabilities	Neurological Disorder
	64	F	W	Administrator for Independent Living Services for People with Disabilities	Polio
	21	M	W	College Student	Mental Health/Learning Disability
University-Affiliated CAB Members	37	F	W	Disabilities and Group and Counseling	Multiple Sclerosis
	55	F	W	*CBPR and Tobacco	N/A
	37	F	W	Tobacco	N/A
	37	M	W	Disabilities and Tobacco	N/A
	31	M	W	Disabilities and Addiction	N/A
	54	F	W	Qualitative Research and Disabilities	N/A
Interviews	54	F	B	Smoker and Community Informant	COPD
	49	M	W	Smoker and Community Informant	Mental Illness
	52	M	B	Smoker and Community Informant	Mobility Impairment
	53	M	B	Smoker and Community Informant	Rheumatoid Arthritis
	58	M	B	Smoker and Community Informant	COPD
	62	F	B	Smoker and Community Informant	Traumatic Brain Injury
	51	M	B	Smoker and Community Informant	Mobility Impairment
	61	M	W	Smoker and Community Informant	Spinal Cord Injury
	54	M	B	Smoker and Community Informant	Mobility Impairment
	56	M	B	Smoker and Community Informant	Mental Illness
Expert Panel	48	M	W	CAB Member	Spinal Cord Injury
	42	F	W	Psychiatrist, Tobacco Research, Cessation, and Mental Illness	N/A
	41	M	W	Physician, Tobacco Cessation, NRT Expert	N/A
	49	M	W	Psychologist, Tobacco Research and Cessation	N/A
	36	M	W	Psychologist, Tobacco Research, Motivational Interviewing	N/A
	51	M	W	*Psychologist, Tobacco Research and Cessation	N/A
	36	F	A	*Psychologist, Tobacco Research and Cessation	N/A

* Denotes the leader(s) of respective stakeholders. “W” denotes White, “B” denotes Black, and “A” denotes Asian.

**Table 2:** Program Recommendations.

	Stakeholders	Action
Logistics		
Program location (at Centers for Independent Living)	I,C*	Incorporated
Increase number of sessions	c	Incorporated
Increase frequency of sessions per week	c	Incorporated
Increase duration of sessions	c	Incorporated
Limit group size to 8 people	c	Incorporated
Account for accessibility challenges (e.g., transportation, scheduling, and changes in health)	I,C	Incorporated
Adjust language requiring all attendees to see a physician prior to participation	E	Incorporated
Manual (Disability Information)		
Content: Use disability-specific examples and scenarios	C, E	Incorporated
Content: Update photos depicting people with disabilities (PWD)	C	Incorporated
Content: Provide more detail on how tobacco use affects disability	I, E	Incorporated
Content: Provide resources for PWD	C, E	Incorporated
Content: Provide disability appropriate recommendations (e.g., incorporate exercises for PWD)	I, C, E	Incorporated
Content: Provide online activities (quizzes, diary)	C	In Progress
Content: Address personal care attendants (PCA) who use tobacco	I, C	Incorporated
Content: Consider how people with cognitive issues track NRT usage	E	Incorporated
Format: Offer alternative formats for hearing and visually impaired	C	Incorporated
Manual (General Accessibility)		
Content: Update general tobacco information (e.g., over 7,000 chemicals are in cigarette smoke)	E	Incorporated
Content: Use appropriate withdrawal symptoms	E	Incorporated
Content: Add more information on stress management	C	Incorporated
Content: Include group rules	C	Incorporated
Content: Use a more flexible quit plan	C,E	Incorporated
Content: Include information and activities on motivation to quit	I, E	Incorporated
Content: Include information on increased appetite and healthy eating	C, E	Incorporated
Content: Add homework assignments	C	Incorporated
Content: Include multiple choice questions within the workbook	C	Evaluating
Content: Remove quit line information from the quit plan page	E	Incorporated
Content: Demonstrate nicotine replacement therapy (NRT)	E	Incorporated
Format: Make information more understandable	C, E	Incorporated
Format: Lower reading level	C, E	Incorporated
Format: Reduce the amount of text / Include more graphics and visuals	C, E	Incorporated
Format: Reduce or remove “scientific” language	C	Incorporated
Facilitator Guide		
Discuss having low familial support in quitting tobacco	C	Incorporated
Discuss challenges of living with a disability and tobacco use (e.g., high number of PWD who smoke)	I, C	Incorporated
Discuss effects of tobacco on wound healing (especially for spinal cord injury)	E	Incorporated
Discuss how medications and NRT interact	I, C, E	Incorporated
Allow participants to select multiple NRT	E	Incorporated
Discuss relapse and slip in proper context	E	Incorporated
Discuss causes of cravings	E	Incorporated
Address questions regarding cessation methods that are not recommended (e.g., e-cigarette, acupuncture)	E	Incorporated
Recommend communication with physician regarding starting program and using medications	E	Incorporated
Provide information regarding handling participants questions about multiple medications	E	Incorporated
Discuss role of caffeine and association with cigarettes	E	Incorporated

* ”I” denotes suggestion made by Individual during qualitative interview; “C” denotes suggestion made by Community Advisory Board Member; “E” denotes suggestion made by expert.
